# Supplementary material for: The causal relationship between the human gut microbiota and pyogenic arthritis: a Mendelian randomization study
Source: Front Cell Infect Microbiol. 2024 Nov 26;14:1452480. doi: 10.3389/fcimb.2024.1452480 (PMC11629706; doi:10.3389/fcimb.2024.1452480)
Supplement: Supplementary file 1 [file DataSheet1.zip › mendelian test/ebi-a-GCST90027634.csv_forest.pdf]

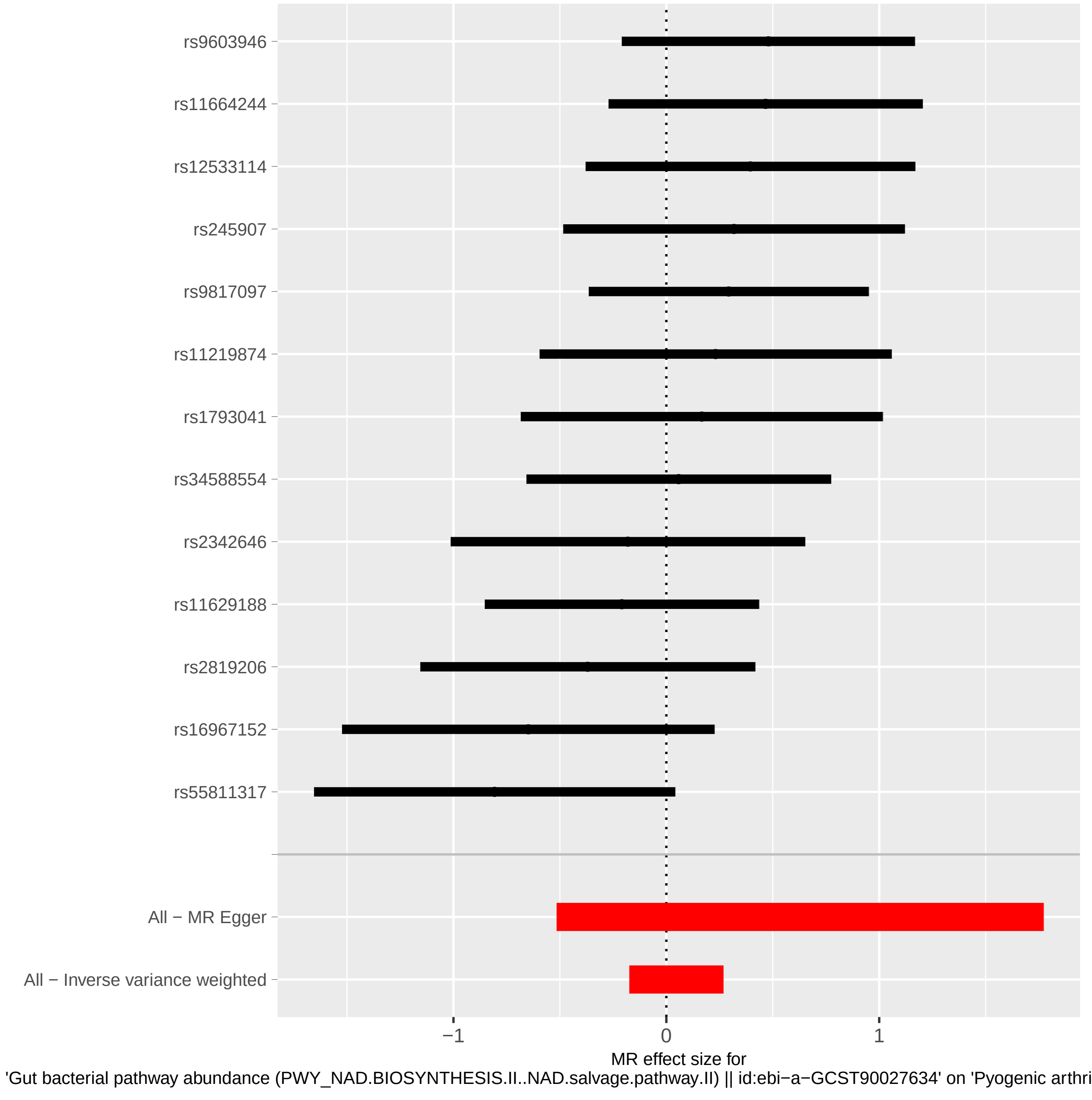

rs9603946
rs11664244
rs12533114
rs245907
rs9817097
rs11219874
rs1793041
rs34588554
rs2342646
rs11629188
rs2819206
rs16967152
rs55811317
All − MR Egger
All − Inverse variance weighted
−1
0
1
MR effect size for
'Gut bacterial pathway abundance (PWY_NAD.BIOSYNTHESIS.II..NAD.salvage.pathway.II) || id:ebi−a−GCST90027634' on 'Pyogenic arthri
